# Endovascular repair with contralateral external-to-internal iliac artery bypass grafting: a case series

**DOI:** 10.1186/s13104-015-1144-6

**Published:** 2015-05-04

**Authors:** Yasuhiko Kobayashi, Masayuki Sakaki, Takashi Yasuoka, Osamu Iida, Tomoharu Dohi, Masaaki Uematsu

**Affiliations:** Department of Cardiovascular Surgery, Kansai Rosai Hospital, 3-1-69 Inabaso, Amagasaki, Hyogo 660-8511 Japan; Departments of Cardiology, Kansai Rosai Hospital, 3-1-69 Inabaso, Amagasaki, Hyogo 660-8511 Japan

**Keywords:** Endovascular abdominal aortic aneurysm repair, Abdominal aortic aneurysm, Common iliac artery aneurysm, External-to-internal iliac artery bypass

## Abstract

**Background:**

To report a technique of keeping unilateral blood flow in the internal iliac artery in cases of an abdominal aortic aneurysm in achieving successful Endovascular abdominal aortic aneurysm repair using an external-to-internal artery bypass.

**Case presentation:**

6 japanese patients with infra-renal abdominal aortic aneurysms were treated using the retroperitoneal approach via a left (right) paramedian incision followed by an external-to-internal artery bypass. Endovascular abdominal aortic aneurysm repair was conducted on mean postoperative day 29 ± 18 and was performed because the contralateral internal iliac artery, which was not involved in the external-to-internal artery bypass, was treated with a coil embolization. No complications developed during the postoperative follow-up period (17 ± 1.5 months). In all 6 patients, patent grafts were evident on computed tomography angiography scans even after 1–3 months.

**Conclusions:**

Endovascular abdominal aortic aneurysm repair with unilateral internal iliac artery embolization and contralateral external-to-internal artery bypass is feasible with a relatively low risk. It is a safe procedure and reduces the incidence of postoperative complications.

## Background

Endovascular abdominal aortic aneurysm repair (EVAR) is an effective alternative therapy for abdominal aortic aneurysms (AAAs) in potentially high-risk patients such as the elderly, those requiring repeated open abdominal surgery, or those with various complications. However, in cases with a short common iliac artery (CIA) or insufficient landing zone, the stent-graft limb must be extended to the external iliac artery (EIA) for treatment. Various complications, such as buttock claudication (due to ischemia of the IIAs), impotence, and intestinal ischemia, arise [1-4] when blood flow to both internal iliac arteries (IIAs) is lost.

In this study, we report cases of 6 patients who underwent EVAR instead of abdominal surgery for the treatment of AAA. EVAR was performed by maintaining unilateral blood flow in the IIA during external-to-internal artery (E-I) bypass.

## Case presentation

### Cases 1 and 2

**Figure 1:**
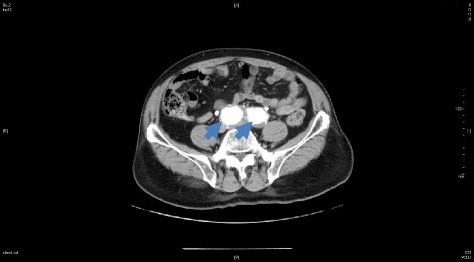
Preoperative CT images of the iliac aneurysm: Case 1.

An 80-year-old Japanese man was referred to our hospital for a urologic disease. Computed tomography (CT) examination of the abdomen showed bilateral CIA aneurysms with a maximal diameter of 40 mm (Figure 1).

A 78-year-old Japanese man was referred to our hospital for AAA. Follow-up was conducted with an annual CT. The patient underwent EVAR after the latest follow-up because the maximal diameter of the AAA was increasing in size and had reached approximately 40 mm, and bilateral CIA aneurysms had maximal diameters of 31–40 mm.

Both patients with bilateral CIA aneurysms that did not have a suitable landing zone for the standard iliac graft limb and therefore underwent an E-I bypass before EVAR.

### Cases 3 and 4

A 68-year-old Japanese man presented to our hospital for the management of alcoholic cirrhosis. The condition was detected during an abdominal CT performed because of abdominal pain. The CT findings showed an AAA (maximal diameter, 55 mm).

A 74-year-old Japanese man with a history of hepatic cell cancer was found to have an AAA (maximal diameter, 45 mm) upon CT examination of the abdomen.

For both patients, E-I bypass was performed prior to EVAR. The stent-graft limb was extended to the EIA when the CIA was short or the landing zone was insufficient.

### Case 5

An 88-year-old Japanese woman underwent EVAR. At three weeks postoperatively, type I endoleak developed. The left CIA aneurysms were approximately 30 mm and were increasing in size.

### Case 6

A 74-year-old Japanese man was referred to our hospital for AAA. He underwent EVAR because the maximal diameter of the AAA was approximately 60 mm and was increasing in size. This case required right E-I bypass due to a contralateral IIA defect.

Six patients (5 men and 1 woman; age, 77 ± 6.8 years) with infra-renal AAAs (largest diameter, 51 ± 5 mm) were included in this study. Patient characteristics are listed in Tables 1 and 2. The AAAs were treated by EVAR using the retroperitoneal approach via the left (right) paramedian incision after an E-I bypass (5 patients, left E-I bypass; 1 patient, right E-I bypass). Subsequently, EVAR was performed using Excluder Gore-Tex (WL Gore and Associates, Flagstaff, AZ, U.S.A) in 4 patients and Zenith Cook (William Cook Europe, Biaeverskow, Denmark) in 2 patients.

**Table 1:** **Patient characteristics**

**Case**	**Age (yrs)**	**Sex**	**Diagnosis**	**Reson for the application of the technique**
1	80	Male	Bi	Without a suitable landing zone for iliac graft limb
2	78	Male	AAA + Bi	Without a suitable landing zone for iliac graft limb
3	68	Male	AAA	CIA is short or the landing zone is insufficient
4	74	Male	AAA	CIA is short or the landing zone is insufficient
5	88	Female	AAA	After EVAR type I endoleak
6	74	Male	AAA	Contralateral IIA defect

AAA: Abdominal aortic aneurysm, CIA: Common iliac artery, EVAR: Endovascular repair, IIA: Internal iliac artery, Bi: bilateral CIA.

**Table 2:** Maximal diameter of aneurysm, follow-up period and outcome

**Case**	**Diameter, (; mm) (L:R ; mm)**	**Follow-up, (mo)**	**Outcome**
1	Bi (40 : 40)	18.1	Survival
2	AAA (38) + Bi (31 : 22)	19.2	Survival
3	AAA (55)	18.5	Survival
4	AAA (45)	10.3	Survival
5	AAA (after EVAR) + LCIA(30)	19	Survival
6	AAA (60)	18	Survival

AAA: Abdominal aortic aneurysm, LCIA: Light Common iliac artery, EVAR: Endovascular repair, Bi: bilateral CIA.

Patients with CIA aneurysms (≥20 mm) without a suitable landing zone (length ≥20 mm) were treated with an aortic cuff or the bell-bottom technique. [5] Patients in whom this method could not be performed were treated with either IIA embolization or extension of the endograft limb into the EIA or advancement of the CIA bifurcation via the E-I bypass. E-I bypass with EVAR was performed for CIA aneurysms (2 patients), short CIAs (i.e., a short landing zone; 2 patients), type Ib endoleak (1 patient), and unilateral IIA defect (1 patient). In the patients who underwent EVAR, IIA aneurysm was not observed; however, varying degrees of IIA sclerosis was found in 1 patient.

The operation was performed via the retroperitoneal approach through a paramedian abdominal incision, and the EIAs and IIAs were exposed. After systemic heparinization, the IIA was clamped proximally and distally, and an end-to-side anastomosis was achieved using a 5–0 polypropylene suture and an 8-mm HemaShield graft (Boston Scientific, Natick, Mass). Similarly, the EIA was clamped proximally and distally, and an end-to-side anastomosis was achieved using a 5–0 polypropylene suture. Proximally, the IIA was ligated to prevent retrograde perfusion. After the distal IIA was mobilized, the 8-mm graft was sutured end-to-side to the artery, and a conduit was sutured end-to-side to the EIA. Distal anastomosis to the IIA was performed, followed by proximal anastomosis in the inferior aspect of the EIA at a sufficient distance from the original bifurcation of the CIA at approximately 2–3 cm. The proximal IIA was ligated to prevent retrograde perfusion, and the graft was anastomosed to the EIA. The mean surgical operative time was 111 ± 35 min, and the total volume of blood loss was 127 ± 99 mL.

We performed EVAR on postoperative day 29 ± 18 in 5 patients. After E–I bypass, coil embolization was performed at the contralateral side to the IIA. The IIA was embolized at its main trunk if no aneurysmal disease was detected. In patients with IIA aneurysmal disease, branches of the IIA were embolized because the postoperative retrograde collateral blood flow into the IIA aneurysm was obstructed. However, bilateral IIA embolizations were not performed. Finally, the patients underwent EVAR, with the bilateral endograft limbs extended into the EIAs at least 2 cm distal to the iliac bifurcations.

**Figure 2:**
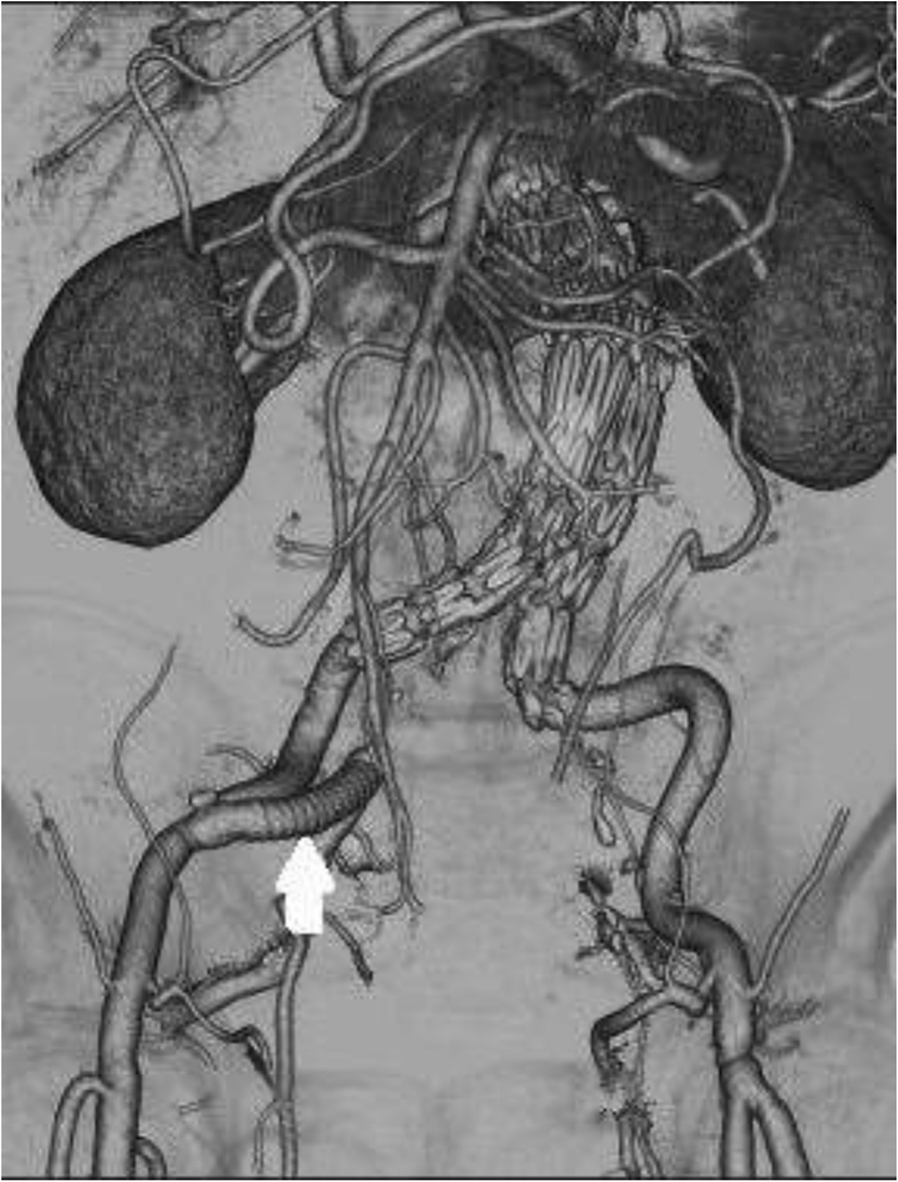
Right external to internal iliac artery bypass was performed using an 8-mm graft (Advanta™ VXT PTFE Vascular Graft). After the internal iliac artery was proximally ligated at its origin to prevent retrograde perfusion, the graft anastomosis was clipped at the external iliac artery.

Postoperative CT angiography (CTA) was performed 1 week after EVAR (Figure 2), and the patients were discharged at 8 ± 1.2 days after their procedure.

No complications, including claudication or intestinal ischemia, developed in the patients during the postoperative follow-up of 17 ± 1.5 months, and the E-I graft in all patients was evident at 1 to 3 months after CTA.

## Discussion

Performing EVAR with E-I bypass concomitantly operation is preferable [6]. Meanwhile, several reports have revealed the feasibility and efficacy of unilateral IIA embolization followed by simultaneous EVAR and contralateral EIA to IIA bypass grafting [7,8]. In general, EVAR with E-I bypass is performed as staged operations in our hospital; however, this is not the intentional result of a policy. In the Department of Cardiology, the treatment department has performed EVAR based on a main diagnosis but has often been unable to perform EVAR with E-I bypass with cardiologists on the same day because of inadequate time. Therefore, EVAR with E-I bypass have also been performed in our hospital as staged operations.

CIA aneurysms occur concomitantly with AAAs in 18% to 25% patients [9,10], and stent-graft limbs are used to block both IIAs when CIAs are treated with EVAR. If the CIA is short, then the landing zone of the contralateral graft limb will be insufficient, and the IIA might be obstructed.

Coil embolization must be applied to both IIAs to prevent the occurrence of a type II endoleak, as well as the possible onset of complications caused by emphraxis of the IIA. One previous report indicated that usually no complications occur, even in cases in which both IIAs are embolized [11]. However, several other studies have disputed this finding and have reported the disruption of pelvic blood flow and other complications such as buttock claudication (26%–41%), impotence (4%–12%), and intestinal ischemia (1%–4%) [1,2].

During conventional replacement with an artificial blood vessel, at least one IIA should be preserved or reconstructed to avoid postoperative complications. Recent reports describing EVAR have indicated the IIA should be reconstructed if it has been sacrificed during the procedure [12,13]. There are two primary methods of reconstructing the IIA. One method involves applying stent grafting using a catheter to treat AAAs [14], while the other method involves a surgical bypass operation [6,12]. The use of both methods has been variously reported.

With respect to stent grafts, Melas *et al*. [15] reported a revised classification to assist in endovascular repair for isolated CIA aneurysms. In the present report, however, there were cases in which stent-graft adaptation would have been unreasonable and in which the long-term outcome remains unclear.

Recently, a branched stent-graft device has become available [16,17]. The iliac branch-graft device (IBD) has shown high technical success rates and encouraging mid-term patency rates; however, this device is not yet common worldwide. More advanced stent-graft devices will appear, and procedures will improve.

From our experience and other reports [12,18], EVAR with unilateral IIA embolization and contralateral E-I bypass is generally feasible. Using a minimally invasive reduction possible, E-I bypass with a retroperitoneal approach might be an effective procedure for preventing retrograde perfusion.

EVAR with unilateral IIA embolization and contralateral E-I bypass is feasible and is associated with a relatively low risk of complications. EVAR with a branched iliac bifurcation device will improve the procedures and will become widely adopted, and thus, will become part of general treatment; however, currently, the technique reported here may be an alternative for treating AAAs.

## Conclusions

Endovascular abdominal aortic aneurysm repair with unilateral internal iliac artery embolization and contralateral external-to-internal artery bypass is feasible with a relatively low risk. It is a safe procedure and reduces the incidence of postoperative complications.

## Consent

Written informed consent was obtained from the patients for publication of this case report and accompanying images. A copy of the written consent is available for review by the Editor-in-Chief of this journal.

## Abbreviations

IIA: Internal iliac artery
EIA: External iliac artery
AAA: Abdomina artery aneurysm
EVAR: Endovascular abdominal aortic aneurysm repair
E–I bypass: External to internal iliac artery bypass
CIA: Common iliac artery
CTA: Computed tomography angiography
